# From Negative to Positive Diagnosis: Structural Variation Could Be the Second Mutation You Are Looking for in a Recessive Autosomal Gene

**DOI:** 10.3390/jpm12020212

**Published:** 2022-02-03

**Authors:** Ioanna Pyromali, Nesrine Benslimane, Frédéric Favreau, Cyril Goizet, Leila Lazaro, Martine Vitry, Paco Derouault, Franck Sturtz, Corinne Magdelaine, Anne-Sophie Lia

**Affiliations:** 1 Faculty of Medicine, MMNP (Maintenance Myélinique et Neuropathies Périphériques), University of Limoges, EA6309, F-87000 Limoges, France; ioanna.pyromali@unilim.fr (I.P.); nesrine.benslimane@unilim.fr (N.B.); frederic.favreau@unilim.fr (F.F.); franck.sturtz@unilim.fr (F.S.); corinne.magdelaine@chu-limoges.fr (C.M.); 2 Centre Hospitalo-Universitaire (CHU) Limoges, Service de Biochimie et de Génétique Moléculaire, F-87000 Limoges, France; martine.vitry@chu-limoges.fr; 3 Centre Hospitalo-Universitaire (CHU) Bordeaux-GH Pellegrin Tripode, Service de Génétique Médicale, F-33076 Bordeaux, France; cyril.goizet@chu-bordeaux.fr; 4 Centre Hospitalier (CH) de la Côte Basque, F-64100 Bayonne, France; llazaro@ch-cotebasque.fr; 5 Centre Hospitalo-Universitaire (CHU) Limoges, Service de Bioinformatique, F-87000 Limoges, France; paco.derouault@chu-limoges.fr

**Keywords:** Charcot–Marie–Tooth, structural variation, *SH3TC2*, CovCopCan, NGS

## Abstract

Next-generation sequencing (NGS) allows the detection of plentiful mutations increasing the rate of patients getting a positive diagnosis. However, while single-nucleotide variants (SNVs) or small indels can be easily detected, structural variations (SVs) such as copy number variants (CNVs) are often not researched. In Charcot–Marie–Tooth disease (CMT), the most common hereditary peripheral neuropathy, the *PMP22*-duplication was the first variation detected. Since then, more than 90 other genes have been associated with CMT, with point mutations or small indels mostly described. Herein, we present a personalized approach we performed to obtain a positive diagnosis of a patient suffering from demyelinating CMT. His NGS data were aligned to the human reference sequence but also studied using the CovCopCan software, designed to detect large CNVs. This approach allowed the detection of only one mutation in *SH3TC2,* the frequent p.Arg954*, while *SH3TC2* is known to be responsible for autosomal recessive demyelinating CMT forms. Interestingly, by modifying the standard CovCopCan use, we detected the second mutation of this patient corresponding to a 922 bp deletion in *SH3TC2* (Chr5:148,390,609-Chr5:148,389,687), including only one exon (exon 14). This highlights that SVs, different from *PMP22* duplication, can be responsible for peripheral neuropathy and should be searched systematically. This approach could also be employed to improve the diagnosis of all inherited diseases.

## 1. Introduction

The diagnosis of inherited genetic diseases to which several causative genes have been associated is usually performed using the targeted next-generation sequencing (NGS) technique. Thus, a disease-specific gene panel is used to detect genomic mutations, improving the rate of patients getting positive diagnosis. Bioinformatic analysis for the detection of variants consists in comparing the patient’s sequences with those of reference sequences from human genome (GRCh37 or GRCh38). However, to date, most of the detected mutations by targeted NGS are SNVs or small indels, but the SVs are often underdiagnosed. This is probably because few user-friendly tools (ExomeDepth, IonCopy, Cov’Cop, CovCopCan, DeviCNV) were available for geneticists to identify the SVs until recently and because these existing tools are maybe not systematically used in routine NGS analysis nowadays [1,2,3,4,5].

Charcot–Marie–Tooth disease (CMT) is the most frequent peripheral hereditary neuropathy, with a prevalence of 1:2500 worldwide, affecting both sensory and motor peripheral neurons and for which broadly two main types have been described based on electrophysiological measurements: CMT1 and CMT2. CMT1 (also known as the demyelinating form) is characterized by reduced nerve conduction velocities (NCV), whereas for CMT2 (called the axonal form) patients present normal NCV values. CMT patients usually manifest progressive distal neuropathy resulting in weakness and atrophy of limb muscles, weak ankle dorsiflexion, depressed tendon reflexes, foot deformities (e.g., *pes cavus*), and distal sensory loss, among others [6]. Symptom severity can differ among patients and disease onset can vary from early childhood to late adulthood. More than 70% of dominant CMT1 cases are explained by the duplication of *PMP22*, which was the first described mutation associated with the disease [7,8,9,10,11]. Currently more than 90 causative genes have been associated with CMT, but the majority of described mutations on these genes are small nucleotide variants (SNVs) or small indels. Only a few structural variations (SVs) have been described in CMT-associated genes, such as in *GJB1*, *MPZ*, *MFN2*, *GAN*, *SEPT9*, *FGD4*, *GDAP1*, *LRSAM1*, *INF2*, *PRX*, *NDRG1*, *MTMR2,* and more recently in *KIF5A* [12,13].

*SH3TC2* codes for SH3 domain and tetratricopeptide repeat-containing protein 2 (SH3TC2), a protein expressed in Schwann cells of peripheral nerves (ensembl: ENSG00000169247, Uniprot-KB: Q8TF17). This protein presents two N-terminal SH3 (Src homology-3) domains and ten tetratricopeptide repeat domains (TPR) arranged in tandem arrays [14]. SH3TC2 localizes to the plasma membrane of Schwann cells and has a role in maintaining the integrity of the node of Ranvier in peripheral nerves and in myelination [15]. SH3TC2 has also been reported localized to the intracellular endocytic recycling compartment, where it regulates the recycling of internalized membrane and receptors by associating with the small GTPase Rab11 [16]. Homozygous or compound heterozygous mutations in *SH3TC2* have been associated with CMT disease following an autosomal recessive transmission mode (OMIM: #601596) [14]. CMT patients harboring mutations in *SH3TC2* are characterized by the presence of severe spine deformities (such as scoliosis) and foot deformities (*pes cavus*, *pes planus*, or *pes valgus*) that typically present in childhood or early adolescence, and they can also present deafness [17,18]. *SH3TC2* is the most frequently mutated gene in patients with recessive demyelinating CMT, with a prevalence of approximately 18% [17,19]. According to Inherited Neuropathy Variant Browser (https://neuropathybrowser.zuchnerlab.net/#/ (accessed on 7 October 2021)), more than 100 different mutations causing CMT have been described in *SH3TC2*. Some of them are more frequent among certain ethnic groups, while c.2860C > T, p.Arg954* mutation is generally the most common mutation, estimated by several studies at 62% of all the SH3TC2 mutations [18,20,21,22].

Herein, by a variant calling analysis of the targeted-NGS data of a patient presenting with severe CMT1 symptoms, a nonsense mutation (p.Arg954*) in *SH3TC2* in a heterozygous state was identified. Then, thanks to the user-friendly CovCopCan software [4], we investigated and detected the deletion of only a single exon in *SH3TC2* presented in a heterozygous state in the same patient. We suggest to regularly use such software to analyze NGS data in order to detect not only large SVs but also small SVs, such as single-exon variants, in order to improve patient diagnosis.

## 2. Materials and Methods

### 2.1. Patients

The proband, a 20-year-old man, presented with demyelinated CMT. His parents were healthy. Peripheral blood was collected into EDTA tubes after informed consent was obtained, and DNA extraction was performed using standard methods (Illustra-DNA-Extraction-kit-BACC3, GEHC). Clinical examination was performed and the Medical Research Council (MRC) Scale for Muscle Strength was used to assess muscle strength from Grade 5 (normal) to Grade 0 (no visible contraction) [23].

### 2.2. Next-Generation Sequencing (NGS) and Bioinformatics Analysis

A 93-gene custom panel designed for diagnosis of CMT and associated neuropathies (as described by [24]) was used for NGS. The amplified library was prepared with Ion-P1-HiQ-Template-OT2-200 kit (Ampliseq-Custom; Life Technologies, Carlsbad, CA, USA), sequenced on Ion-Proton sequencer (Life-Technologies), and mapped to the human reference genome GHCh37. Alamut Visual Interpretation Software v.2.11 (Interactive Biosoftware, Rouen, France), using the NM_024577.4 reference sequence for the *SH3TC2* gene was used to evaluate the variants. Databases such as gnomAD (https://gnomad.broadinstitute.org/ (accessed on 14 October 2021)), dbSNP135 (National Center for Biotechnology Information [NCBI], http://www.ncbi.nlm.nih.gov/projects/SNP/ (accessed on 14 October 2021)) and Clin-Var (www.ncbi.nlm.nih.gov/clinvar (accessed on 14 October 2021)) were also used.

SVs were detected using CovCopCan software starting from the coverage file provided after NGS by Ion-Proton sequencer [4]. In brief, CovCopCan software uses a two-stage correction and normalization algorithm to identify unbalanced SVs, such as CNVs (copy number variants) using NGS read depth.

### 2.3. Verification of Mutations

PCR and Sanger sequencing experiments were performed in order to verify the presence of the pointed mutation detected by the variant calling analysis of NGS data. The primers used were ACTCCAAGGTGAAGGCCGG and TAGAAATGGCAGAGGGATTTG.

Regarding the deletion, long-range PCR and Sanger sequencing experiments were used in order to define the exact breakpoints of the deletion. For the PCR experiments, the Master Mix Phusion Flash (Thermo Scientific, Waltham, MA, USA) and the following primers (Sigma-Aldrich, St. Louis, MO, USA) were used: primer Int12-F: GCTGTTCCTGCTCAGAGCTT in intron 12 and Int15-R: CACACCCAATAGTGAAGACCA in intron 15. Sanger sequencing experiments were performed on PCR products by a walking primer strategy and the Big Dye Terminator Cycle Sequencing Kit v2 (ABI Prism, Applied Biosystems, Waltham, MA, USA). The exact breakpoints were identified thanks to primers (Sigma-Aldrich) Int13-F: AGGATTCCATCTCACTGCC in intron 13 and Int14-R: CTGAGATGGTCTTGATCTCC in intron 14. 

## 3. Results

### 3.1. Patient’s Clinical Description

Our current study focuses on the family of a single affected member. The propositus presented with a severe demyelinating neuropathy confirmed by electromyography (Table 1). Muscle testing, using the Medical Research Council (MRC) Scale for Muscle Strength revealed a deficit in all four limbs, predominantly in the lower limbs and essentially in the levators and evertors of the feet, with values of 3/5 [23]. There was also an impairment in the upper limbs with a muscular testing at 4/5 proximal and 3/5 in the hands. The patient’s osteotendinous reflexes were abolished. He presented with a bilateral equinus also with a varus position and bilateral hollow feet that were painful. Achilles tendons retraction was present, leading to difficulties in standing without moving. He wore bilateral over-pedal splints to improve his walking and wore a corset to correct his scoliosis. Additionally, he had trouble in fine motor control and digital dissociation and a lack of strength, which did not impair his activities in daily life. 

**Figure 1:**
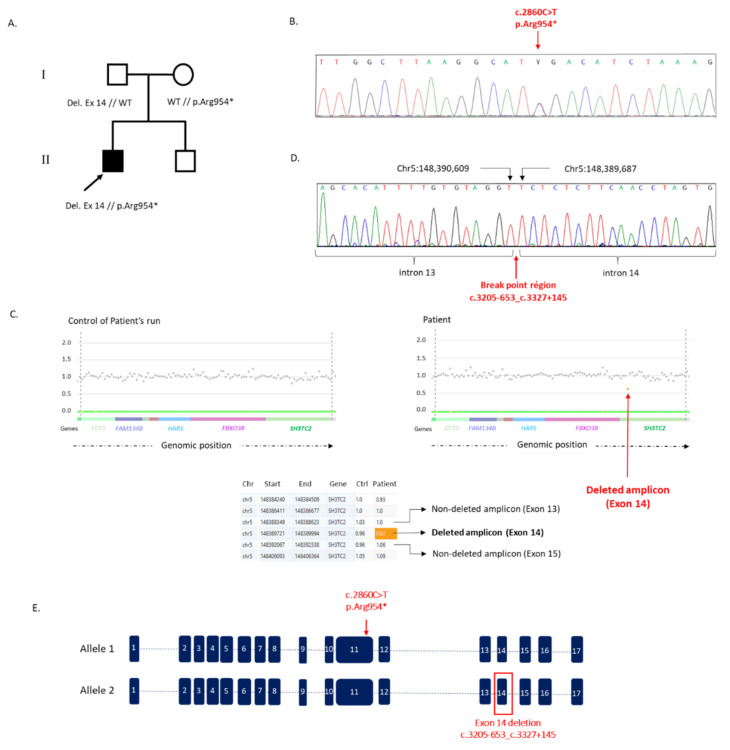
Family’s pedigree, analysis of *SH3TC2* by CovCopCan and verification of mutations by Sanger sequencing. (**A**) Family’s pedigree. WT indicates the normal *SH3TC2* allele, “p.Arg954*”, the allele presenting the missense mutation c.2860C > T leading to a premature terminal codon and Del ex14, the allele with *SH3TC2* deletion corresponding to c.3205-653_c.3327 + 145 del including exon 14. (**B**) Sanger sequencing of the first mutation c.2860C > T, p.Arg954* at the heterozygous state. (**C**) Graphical representation and table extracted from CovCopCan analysis for the patient and for a control (Ctrl). In the graphical representation, each dot represents an amplicon. Normal amplicons (in gray) have values around 1, whereas deleted amplicons (in orange) have values around 0.5. Deleted amplicon corresponds to *SH3TC2* exon 14. Start and end on the table correspond to the chromosomic positions of amplicons. (**D**) Detection of breakpoints in *SH3TC2* by Sanger sequencing. The breakpoints were localized in position Chr5:148,390,609 in intron 13 and in position Chr5: 148,389,687 in intron 14. (**E**) Schematic overview of *SH2TC2* and variations localization. Blue boxes correspond to *SH3TC2* exons.

Regarding the sensory aspect, he presented a dysesthesia localized in the distal part of the lower limbs, with unpleasant tingling sensations either after hot shower or when immobile. In addition, he presented with disorder of superficial bilaterally, epicritic sensitivity, associated with proprioceptive disorder. Nevertheless, he had good superficial sensitivity in the proximal part of the lower limbs and in the upper limbs. The symptoms appeared in early childhood, and he was the only member of his family presenting such symptoms (Figure 1A).

### 3.2. Detection of SNPs and Structural Variants

Patient’s DNA was analyzed by NGS using a 93-gene panel involved in peripheral neuropathies [24]. The standard alignment bioinformatic analysis of the NGS data revealed the presence of a unique mutation in *SH3TC2*, the known mutation c.2860C > T, p.Arg954*, leading to the appearance of a premature termination codon (Figure 1B). This mutation, initially described by Senderek et al. in 2003 [14], appeared to be the most frequent mutation in *SH3TC2* gene, estimated recently to 62% of the mutations in a French *SH3TC2* cohort [18]. With *SH3TC2* being associated with a CMT form transmitted by autosomal recessive mode, this mutation could not explain by itself the severe symptoms of the patient [14]. However, no additional SNV or short indel was detected. In parallel, the standard use of CovCopCan, which easily highlights CNVs when at least three successive amplicons are deleted or duplicated, did not allow the detection of any CNVs in the 93 genes tested in the first place. Nevertheless, following the discovery of the point mutation in *SH3TC2*, we modified and personalized the use of CovCopCan by looking more specially for deletion or duplication in *SH3TC2* involving less than three amplicons. This new approach allowed pointing out a potential deletion of one amplicon covering the genomic region Chr5:148,389,721-Chr5:148,389,994 (Figure 1C). According to CovCopCan, this deletion would be in a heterozygous state and would correspond to the deletion of *SH3TC2* exon 14, whereas amplicons corresponding to exons 13 and 15 were not deleted.

### 3.3. Confirmation of the Structural Variant and of the Nonsense Mutation

A long-range PCR, using primers located on the non-deleted exons 13 and exon 15, was performed in order to confirm the presence of the potential deletion pointed out by CovCopCan software and allowing the detection of two bands, confirming the presence of the deletion in one allele (not shown). Sanger sequencing experiments were then conducted on the lower band in order to identify the exact breakpoint positions at Chr5:148,390,609 in intron 13 and Chr5:148,389,687 in intron 14 (Figure 1D), corresponding to a 922 base pairs deletion. The breakpoints of this *SH3TC2* deletion were located in intron 13 at position c.3205-654 and in intron 14 at position c.3327 + 146, leading to the mutation c.3205-653_c.3327 + 145 del (p.Ala1069_Arg1109 del), which corresponded to the deletion of exon 14 (Figure 1E). In addition, the c.2860C > T, p.Arg954* was confirmed by PCR and Sanger sequencing (Figure 1B). Molecular analyses of the unaffected parents confirmed the compound heterozygous status of the proband.

## 4. Discussion

Herein, by analyzing NGS data of a patient presented with a severe demyelinating neuropathy, we detected the presence of the known nonsense mutation (c.2860C > T, p.Arg954*) in heterozygous state thanks to standard variants detection software. However, this mutation alone was not enough to explain the severity of the patient’s phenotype [14]. Thus, by modifying the use of the user-friendly software CovCopCan, we achieved the detection of a SV in *SH3TC2* in the second allele, corresponding to a deletion of exon 14. The presence of this deletion was confirmed, and the breakpoints were identified at positions Chr5:148,390,609 and Chr5:148,389,687, corresponding to a 922 bp deletion that may have led to the mutation c.3205-653_c.3327 + 145 del (p.Ala1069_Arg1109 del). Thus, this new approach allowed achieving the patient’s correct diagnosis.

Patients harboring two mutations in *SH3TC2* present with severe neuropathy of the peripheral nervous system characterized by important decrease in NCVs, severe spine and foot deformities, and sometimes also a cranial nerve involvement (manifested by hearing impairment and facial weakness among others); symptoms usually appeared in the first decade of life [17,18]. The patient described in this paper presented a severe demyelinating neuropathy as well, associated with a motor and sensory disorder on the four limbs, severe foot deformities, and scoliosis since childhood, confirming the fact that the two detected mutations in *SH3TC2* were certainly the cause of his disease.

Regarding the effect of these mutations, our patient presented the nonsense mutation p.Arg954* that could lead to the production of a truncated protein. In addition, he harbored a newly described SV leading to the deletion of exon 14 in *SH3TC2*. For this allele, if the splicing of the truncated mRNA was performed correctly between exon 13 and exon 15, the protein’s translation would remain in phase. However, the deleted area would correspond to the final part of the tetratricopeptide repeat functional domain that would potentially alter SH3TC2 function [16]. Another possibility would be that the truncated mRNA would not be spliced correctly between exon 13 and exon 15 and would generate an aberrant mature mRNA that would lead to a non-functional SH3TC2 protein. Nevertheless, with *SH3TC2* being expressed mainly in Schwann cells, it is currently not possible to arrive at conclusions due to the lack of nervous tissue availability [15].

It is important to underline that, in this case, the small SV involving a single exon was detected only because there was already a nonsense mutation detected on the heterozygous state in *SH3TC2* in the patient that led us to look meticulously for a second mutation in order to explain the patient’s symptoms. Indeed, we modified the use of CovCopCan by looking particularly for small deletion or duplication in *SH3TC2*. It is interesting to notice that software classically detecting CNVs from amplicons sequencing usually recommend the research of three successively deleted or duplicated amplicons in order to highlight with certainty real CNVs [1,2,3,4,5]. Indeed, while bioinformatic tools for CNVs detection broadly have a high specificity (96% for CovCopCan), false positives may occur, and thus it is not possible to routinely study every single deleted or duplicated amplicon. However, we proved here that this “classic” strategy has to be slightly modified in some cases. We suggest that, for patients for whom only one mutation is identified while two mutations are expected (whatever the gene), it would be interesting to check thoroughly the NGS analysis and verify whether a single amplicon deletion or duplication is present on the same gene. We believe that this approach could be performed for all NGS analyses, whatever the inherited studied diseases, in order to improve diagnosis.

## 5. Conclusions

In conclusion, we showed that the newly described exon 14 deletion associated with the nonsense c.2860C > T, p.Arg954* in *SH3TC2* was responsible for the demyelinating CMT disease. This small deletion was identified thanks to a slightly modified use of CovCopCan software, a user-friendly software analyzing NGS data by using the reads’ depth [4]. It is important to underline that SV of a single exon deletion type may cause or contribute to the appearance of a patient’s symptoms and that software able to detect the deletion of a single exon should be widely used in order to improve a patient’s diagnosis.

## Figures and Tables

**Table 1:** Patient’s neurophysiological recordings. Abnormal values are represented in bold. Normal values of MNCV: ulnar (>52 m/s; >7.9 mV), median (>49 m/s; >4.1 mV), tibial ((>39 m/s; >4.4 mV), fibular (>43 m/s; >1.3 mV) [25]. (Vel: velocity; Amp: amplitude; NR: no response).

Motor Nerve Conduction Values (MNCV)									
Ulnar		Median		Tibial		Fibular Right		Fibular Left	
Vel (m/s)	Amp (mV)	Vel (m/s)	Amp (mV)	Vel (m/s)	Amp (mV)	Vel (m/s)	Amp (mV)	Vel (m/s)	Amp (mV)
20.7	2.9	26.2	0.97	NR	NR	22.1	0.23	21.0	0.23

## Data Availability

In this section, please provide details regarding where data supporting reported results can be found, including links to publicly archived datasets analyzed or generated during the study. Please refer to suggested Data Availability Statements in section “MDPI Research Data Policies” at https://www.mdpi.com/ethics (accessed on 7 January 2022). You might choose to exclude this statement if the study did not report any data.
